# Effect of Degree of Ethoxylation on the Surface and Thermal Properties of Polymeric Ionic Liquids for Oilfield Applications

**DOI:** 10.3390/polym17050580

**Published:** 2025-02-22

**Authors:** Mohammed Alotaibi, Mohanad Fahmi, Masooma Nazar, Ahmad Mahboob, Syed Muhammad Shakil Hussain, Muhammad Shahzad Kamal

**Affiliations:** 1 EXPEC Advanced Research Center, Saudi Aramco, Dhahran 31311, Saudi Arabia; mohammad.otaibi.61@aramco.com (M.A.); mohanad.fahmi@aramco.com (M.F.); 2 Center for Integrative Petroleum Research, College of Petroleum Engineering and Geosciences, King Fahd University of Petroleum and Minerals, Dhahran 31261, Saudi Arabia; masooma.nazar@kfupm.edu.sa (M.N.); ahmad.mahboob@kfupm.edu.sa (A.M.)

**Keywords:** enhanced oil recovery, polymeric ionic liquids, surface properties, thermal stability

## Abstract

Worldwide energy needs are growing, requiring new extraction techniques for crude oil from old reservoirs. However, conventional chemicals face difficulties when exposed to harsh reservoir environments such as solubility in high saline water and heat stability under harsh reservoir environments. This study investigates the potential of newly synthesized polymeric ionic liquids (PILs) as alternative options. A series of PILs was synthesized and characterized by using NMR and FTIR techniques. It was noticed that a PIL without ethoxy groups exhibits precipitation and therefore is not suitable for oilfield applications. However, the incorporation of ethoxy groups in the chemical structure of PILs leads to excellent solubility in low to high salinity brine. The solubility of the synthesized PILs in formation water, seawater, and deionized water, as well as their thermal stability using thermal gravimetric analysis (TGA), was assessed. In addition, the surface properties, including critical micelle concentration (cmc), surface tension (*γ_cmc_*), surface excess concentration (*Γ_max_*), minimal surface area per molecule (*A_min_*), free adsorption energy (*ΔG*°*_ads_*), and free micellization energy (*ΔG*°*_mic_*), were also evaluated. The findings revealed that adding ethoxy groups in PILs led to a drop in *Γ_max_* and an increase in *A_min_*, suggesting reduced monolayer compactness at the air/water interface. The synthesized PILs demonstrated remarkable solubility, heat stability, and resistance to salt, rendering them well-suited for oilfield applications under challenging reservoir environments.

## 1. Introduction

The production of crude oil from old reservoirs using primary and secondary methods is low, even though the global need for energy is increasing [1,2]. The decreasing rate of new oil field discoveries and increasing global energy demand have necessitated the redevelopment of old oil reservoirs using modern extraction methods [3]. Water flooding in traditional reservoirs often leads to the extraction of 15–20% of the oil until it reaches the point where it is no longer economically viable to extract. Enhanced oil recovery (EOR) technology is necessary to extract the residual oil from reservoirs. Various EOR techniques including chemical flooding, thermal recovery, and miscible gas injection are successfully applied in different reservoirs [4]. Chemical EOR is a prominent approach that involves injecting chemicals to enhance the effectiveness of displacing oil [5,6]. The primary problem with traditional chemical EOR techniques is their degradation under severe reservoir conditions, which can lead to environmental issues [7]. Consequently, the oil and gas industry has been exploring environmentally friendly alternative chemicals that can adapt to the unique needs of reservoirs.

Currently, there is a growing interest in ionic liquids (ILs) because of their remarkable properties [8,9]. ILs are organic compounds of ionic molecules that maintain a liquid state throughout a wide spectrum of temperatures and demonstrate surface activity. Because they have less toxicity, a lower critical micelle concentration (cmc), high thermal stability, are non-corrosive, and are environmentally benign, they have the potential to replace conventional surfactants [10,11,12]. ILs are commonly made up of organic or inorganic anions and organic cations. By modifying the cations or anions, it is possible to obtain specific ILs with the required characteristics [13,14]. Polymeric ionic liquids, a subclass of polyelectrolytes, contain an ionic liquid species in each repeating unit of polymerized IL [15,16]. Numerous research investigations have shown the advantages of ILs for EOR applications. Manshad et al. [17] demonstrated the use of four ILs to modify wettability and reduce interfacial tension (IFT), resulting in a 13% improvement in oil recovery when the optimal IL flooding conditions were used. Nandwani et al. [18] performed a comparison between the usage of IL ([C_16_mim] [Br]) and the commonly utilized Cetyltrimethylammonium Bromide (CTAB) cationic surfactant. It was observed that IL performed more effectively in challenging environments and facilitated oil recovery by reducing the IFT values. In recent work, Esfandiarian et al. [19] investigated the interaction between crude oil and three imidazolium-based ILs-1-hexyl-3-methylimidazolium chloride ([HMIM][Cl]),1-octyl-3-methylimidazolium chloride ([OMIM][Cl]), and 1-dodecyl-3-methylimidazolium chloride ([DMIM][Cl]) at the interface across a broad range of salinities. They observed that the decrease in IFT and emulsification propensity of low saline water was enhanced as a result of the salting-in and saponification impact. Additionally, it was shown that the cmc of ILs reduced as the salinity increased. This suggests that a smaller amount of ILs is needed for EOR applications.

Protic ionic liquids (PILs) are an excellent substitute for classic ILs (aprotic ILs) because aprotic ILs are costly due to their complicated synthesis methods. PILs can be synthesized by using inexpensive chemicals and simple synthesis methods [20]. In PILs, Brønsted base acts as a cation, and Brønsted acid functions as an anion. These PILs possess outstanding characteristics, including their increased conductivity and superior electrochemical capabilities [21]. Furthermore, these compounds easily exchange one or more hydrogen atoms (in the positively charged species), which facilitates the formation of significant hydrogen bonding connections. The majority of studies on ILs have focused on common ILs such as alkyl pyridinium, alkyl imidazolium, or alkylammonium [22,23]. Most investigations on this subject have included comprehensive lists of these ILs [24,25]. However, there have been limited research studies conducted to investigate the properties of ILs that include anions produced from organic acids, specifically for their potential use in EOR applications. In addition, the applications of polymeric ILs for the EOR process are rare in the literature [26].

Polymeric ionic liquids (PILs) are a subclass of ionic liquids that consist of macromolecules while retaining the unique properties of conventional ionic liquids. PILs can be synthesized either through polymerization or by modifying existing ionic polymers [15,27]. Their properties can be tailored to suit specific reservoir conditions. PILs exhibit excellent characteristics, including high thermal stability, tunable structures, lower toxicity compared to other polymers and ionic liquids, and enhanced durability. In chemical-enhanced oil recovery (EOR), PILs combine the stability of ionic liquids at high temperatures and salinity with the flexibility and durability of polymers [28]. They have demonstrated significant potential in modifying wettability, reducing interfacial tension (IFT), and improving rheological properties. However, there is still limited literature on the application of PILs for EOR under high-temperature and high-salinity conditions [26]. Due to these promising properties, researchers have recently begun exploring the potential of PILs for EOR.

In developing and using extended ILs, it was found that they can significantly lower surface tension and have good tolerance to salt water, specific phase behavior, and strong solubilization performance [29]. Choosing the right components within the IL structure is crucial for achieving the desired properties for oilfield applications, as using the wrong ILs can lead to high adsorption and reduced oil recovery [30]. Experiments have shown that the incorporation of ethoxy (EO) groups combines benefits such as good solubility, salt resistance, stability, dispersibility, and compatibility. Additionally, the surface and thermal behavior can be adjusted by varying the number of EO groups [31].

In this study, polyoxyethylene alkyl ether carboxylic acid containing different numbers of polyethylene oxide (EO) units was chemically modified into ionic liquids (PILs) for EOR application [32]. The addition of EO is usually achieved through oxyanionic polymerization of epoxides [33]. However, in our study, we used commercially available Glycolic acid ethoxylate lauryl ether with average M_n_ (360, 460, 690, and 700) containing various EO units. The term “polymeric ionic liquids” used in the manuscript is the relative term highlighting the effect of the incorporation of different polyoxyethylene units in the chemical structure. The chemical structures of the synthesized PILs were confirmed using NMR and FTIR. The salt solubility of these PILs was tested in formation water (FW) seawater (SW) and deionized water (DW). Surface properties such as critical micelle concentration (cmc), surface tension at cmc (*γ_cmc_*), surface excess concentration (*Γ_max_*), and minimum surface area per molecule (*A_min_*) were also investigated. Additionally, thermodynamic parameters such as free adsorption energy (*ΔG*°*_ads_*) and free micellization energy (*ΔG*°*_mic_*) were also examined.

## 2. Materials and Methods

### 2.1. Materials

Glycolic acid ethoxylate lauryl ether (average M_n_~360, 460 and 690), glycolic acid ethoxylate oleyl ether (M_n_~700), and ethanolamine were obtained from Sigma-Aldrich (Waltham, MA, USA). The solvents were purchased from Sigma-Aldrich and used without purification. The salts that are used for the preparation of simulated seawater (SSW) and simulated formation water (SFW) were received from VWR Chemicals BDH (Radnor, PA, USA). The salt composition of SSW and SFW used in this study is mentioned in Table 1.

### 2.2. Synthesis Process of PILs

The PILs were synthesized by the treatment of an equimolar amount of glycolic acid ethoxylate lauryl ether with ethanol amine in a round-bottom flask at ambient temperature. Specifically, the ethanolamine was introduced into the flask, followed by the dissolution of glycolic acid ethoxylate lauryl ether in methanol. The prepared acid was applied slowly and in small drops to the flask, which was placed in an ice bath. The reaction mixture was agitated for 24 h at a temperature of 25 °C. The excess methanol was extracted from the mixture, and the synthesized PILs were kept in a dark environment at room temperature.

### 2.3. Chemical Structure Elucidation

The structures of the synthesized PILs were verified using nuclear magnetic resonance (NMR) analysis conducted with a Jeol 1500 spectrometer (Jeol, Tokyo, Japan). The solvents employed were deuterated chloroform (CDCl_3_) and methanol-D4 (CD_3_OD). Fourier Transform Infrared Spectroscopy (FTIR) was used to find the different functional groups in the material using the PerkinElmer FT-IR instrument 16 F model (Perkin-Elmer, Waltham, MA, USA). The frequency spectra were obtained by scanning in the range of 4000–600 cm^−1^ using 64 scans.

### 2.4. Thermal Gravimetric Analysis

The thermal properties of the prepared PILs were examined using a thermogravimetric analyzer (SDT Q600, New Castle, DE, USA). The samples were analyzed using a continuous flow of nitrogen. The analysis required heating the samples from room temperature to 1000 °C at a rate of 10 °C per minute.

### 2.5. Salt Tolerance Test

Tests for salt solubility were conducted using 0.25 wt% solutions of each PIL in SFW, SSW, and DW. The prepared samples were placed in an oven at 90 °C for 10 days. Throughout this period, the solubility of the PILs was consistently monitored to detect any indications of phase separation, turbidity, or precipitation.

### 2.6. Surface Tension Measurement

The synthesized PILs’ surface tension was measured at ambient temperature using a force tensiometer. The surface tension was precisely measured by the tensiometer made by the Gothenburg, Sweden-based company Biolin Scientific. The Wilhelmy plate was used to determine the surface tension. Before each experiment, the Wilhelmy plate had been rinsed with distilled water and subjected to a blue flame for burning. The surface tension of distilled water was determined as a standard for comparison.

## 3. Results and Discussion

**Scheme 1:**
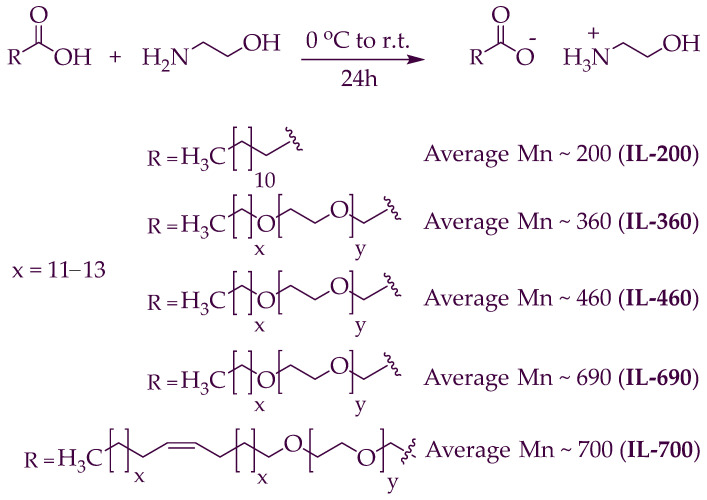
The method for synthesizing polymeric ionic liquids.

The synthesis process for the polymeric ionic liquids (PILs) (PIL-200, PIL-360, PIL-460, PIL-690, PIL-700) is shown in Scheme 1. These PILs were formed by adopting the procedure reported in the literature [30,31]. The developed PILs were dried under a high vacuum for a minimum of 7 h, and no evidence of any byproducts or initial substances was detected in their ^1^H and ^13^C NMR spectra. The yield of synthesized PILs was (99%).

### 3.1. NMR of PILs

**Figure 1:**
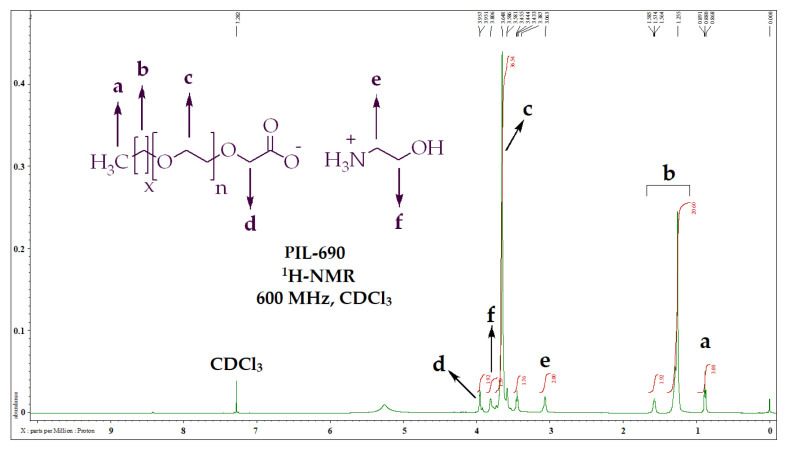
^1^H-NMR data and peaks description of PIL-690.

The chemical structures of the prepared PILs (PIL-200, PIL-360, PIL-460, PIL-690, PIL-700) were established using NMR and FITR spectrometer. The structure analysis of PIL-690 is represented here as an example. Regarding the ^1^H-NMR analysis of PIL-690 (Figure 1, Table 2), the terminal methyl (CH_3_-) was resonated at *δ* 0.88 ppm and the multiple methylenes (-CH_2_-) were detected at *δ* 1.26 ppm. The two methylene groups (-CH_2_) of ethanolamine were resonated at *δ* 3.06 ppm and *δ* 3.81 ppm. The repeating ethoxy groups were spotted at *δ* 3.65 ppm.

**Figure 2:**
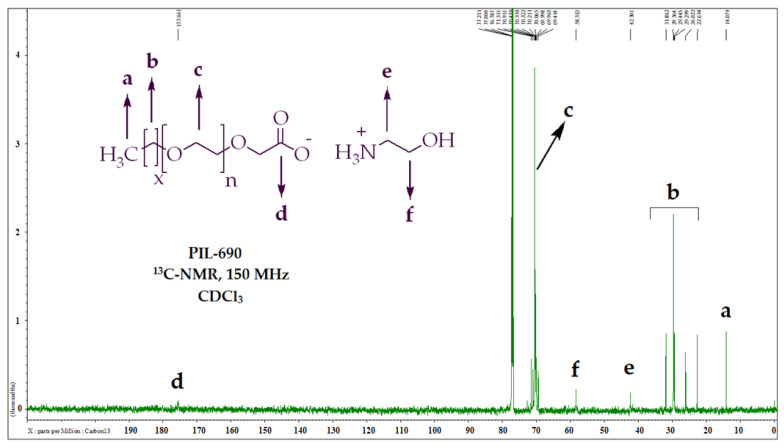
Carbon NMR graph and peaks description of PIL-690.

According to carbon NMR results of PIL-690 (Figure 2, Table 3), the resonating signals at *δ* 14.1 ppm were assigned to terminal methyl (-CH_3_), and the repeating methylenes (-CH_2_) appeared at 22.6–31.8 ppm. The two methylene groups (-CH_2_) of ethanolamine were detected at *δ* 42.3 ppm and *δ* 58.3 ppm. The repeating ethoxy groups were observed at *δ* 70.1 ppm and the carbonyl carbon was resonated *δ* 175.7 ppm.

### 3.2. FTIR of PILs

**Figure 3:**
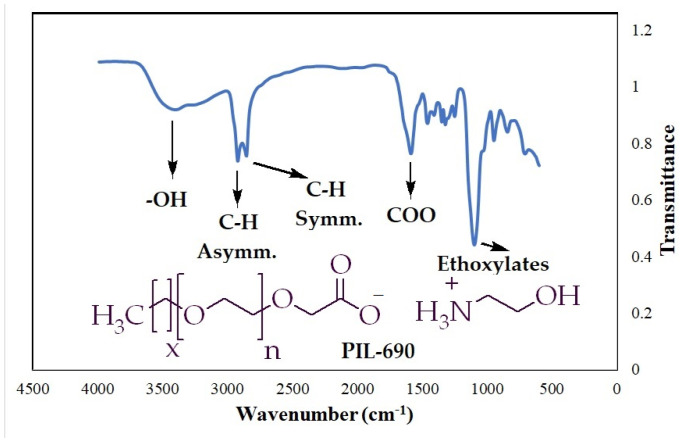
FTIR spectra for the PIL-690.

To confirm the various functionalities in the polymeric ionic liquid (PIL-690), the FTIR technique was conducted, and the results are summarized in Figure 3 and Table 4. The stretching vibration of the O-H bond was observed at 3414 cm^−1^ and the asymmetric as well as symmetric vibrations of aliphatic C-H bond were detected at 2923 cm^−1^ and 2855 cm^−1^, respectively. The strong absorption band at 1585 cm^−1^ was attributed to carbonyl functionality and the highest intensity peak at 1099 cm^−1^ was assigned to repeating ethoxy groups.

### 3.3. TGA Analysis

**Figure 4:**
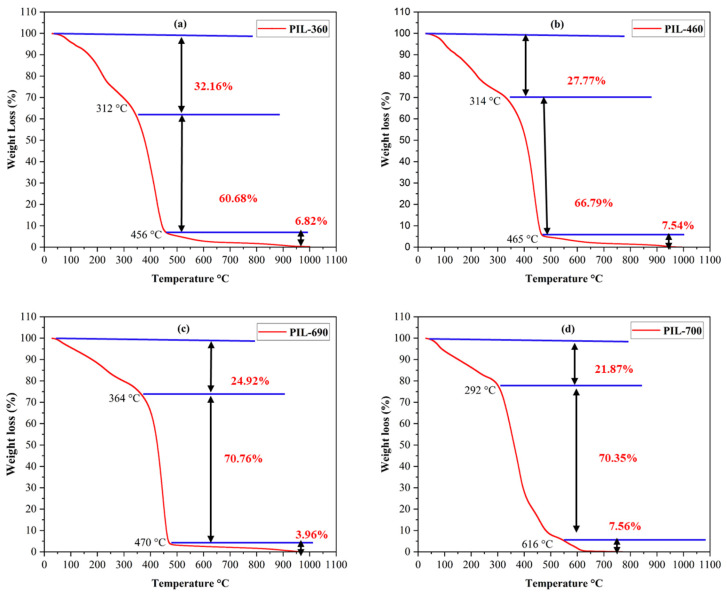
TGA analysis of the PILs at 30 °C–1000 °C: (**a**) PIL 360, (**b**) PIL 460, (**c**) PIL 690, (**d**) PIL 700.

TGA analysis was performed to evaluate the thermal stability of the synthesized PILs, as demonstrated in Figure 4. The thermal stability of the PILs has been shown up to 479 °C for PIL-360, PIL-460, and PIL-690, and 616 °C for PIL-700 (as shown in Figure 4a–d respectively). Three distinct phases of weight loss were observed for every kind of PIL when the temperature was raised. These PILs exhibit little decomposition below 100 °C, gradual decomposition between 110 °C and 360 °C, and fast decomposition until 616 °C. After this, the percentage of weight loss changed relatively less as the temperature increased. The first phase involves the vaporization of any remaining solvent in the PILs, but the composition of the PILs remains unaffected within this temperature zone [34,35]. The weight loss of the produced PILs in the second step was correlated with the breakdown of chemically bound solvents. The breakdown of carbon bonds at high temperatures caused the weight loss to increase in the third and final phase [36,37]. The initial weight loss temperatures for PIL-360, PIL-460, PIL-690, and PIL-700 are 312 °C, 314 °C, 364 °C, and 292 °C, respectively. The PIL-360, PIL-460, PIL-690, and PIL-700 have final temperatures of 456 °C, 465 °C, 470 °C, and 616 °C, respectively, for thermal degeneration. The TGA findings demonstrate that these PILs have exceptional thermal stability which makes them ideal for reservoir environments exceeding 90 °C.

### 3.4. Salt Tolerance of PILs

The majority of chemicals used in chemical floods lose their solubility as the salinity level increases deep down in the reservoir. Chemical solutions form precipitates when mixed with brine due to divalent or multivalent ions, such as calcium (Ca^2+^) and magnesium (Mg^2+^). These ions reduced chemical solubility, leading to aggregation and precipitation. This can make chemical-based flooding processes less effective, which shows how important it is to think about the ionic composition of reservoir brine when choosing any chemical for oilfield applications [38]. Reservoirs with high salinity often have large salt concentrations in the formation water, ranging from 10% to 15% by weight [39]. This study evaluated PILs’ salt tolerance in high-saline conditions to determine their effectiveness in oil recovery processes. The solubility and stability of the PILs were analyzed to assess their compatibility with high-saline reservoirs, which pose challenges for conventional chemicals used for EOR because of their high ionic strength.

**Figure 5:**
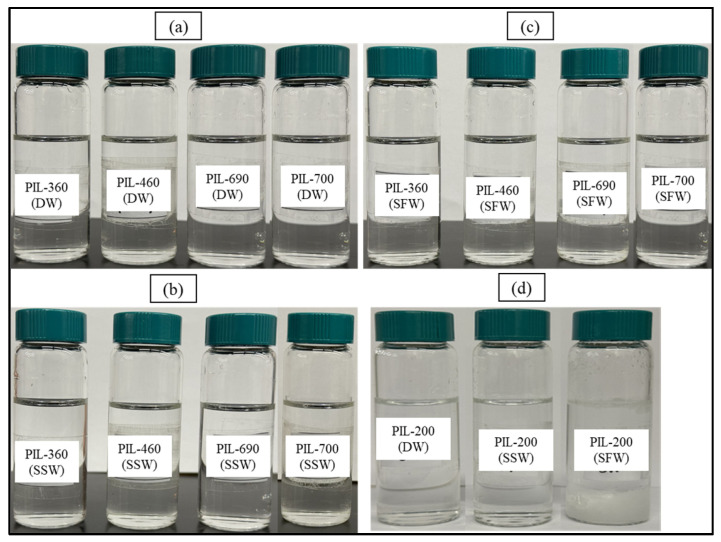
Snapshot of 10-day oven-aged samples at 90 °C: (**a**) PILs in DW, (**b**) PILs in SSW, (**c**) PILs in SFW, and (**d**) PIL-200 in DW, SSW, and SFW.

Figure 5 depicts the evaluation of the PILs’ solubility at 90 °C after 10 days. The current salt tolerance research on PILs revealed no precipitation at 90 °C, even within high salinity brine (SFW). However, PIL-200 with no ethoxy units exhibited precipitation in SSW and SFW. The results of all PILs are demonstrated in Table 5. Hence, the developed PILs exhibit stability in both high-salinity and high-temperature conditions. Each PIL showed excellent solubility efficiency, with no instances of insoluble components found over the whole testing period, despite the use of synthetic brine.

### 3.5. Surface Tension Analysis

Surface tension measurements were conducted to examine the interactions occurring at the interface between water and air. These measurements helped to determine various parameters, such as the critical micelle concentration (cmc), surface tension at the cmc (*γ_cmc_*), the effectiveness of decreasing the surface tension (*Π_cmc_*), maximum surface excess concentration (*Γ_max_*), and the minimum surface area occupied by a molecule at the interface (*A_min_*) for the synthesized PILs using a Wilhelmy plate on a force tensiometer. Table 6 summarizes the surface property values for the PILs used in this study.

**Figure 6:**
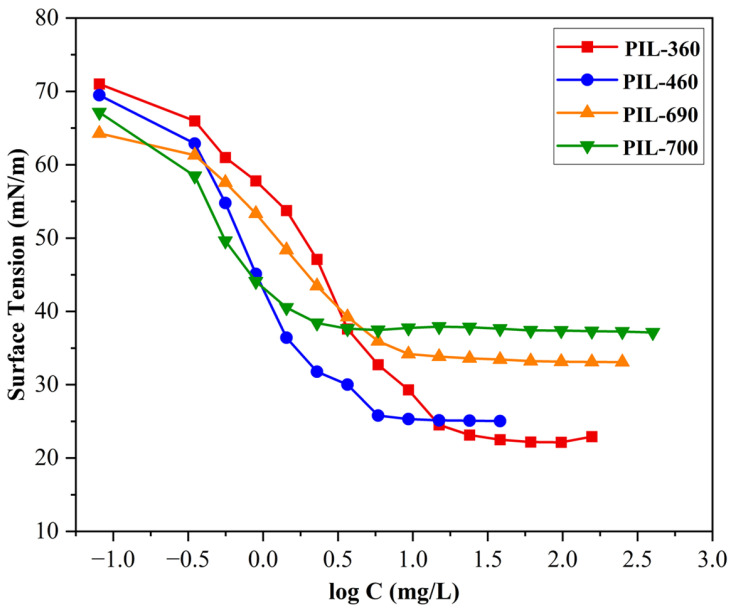
Surface tension vs log C of the synthesized PILs at room temperature.

Figure 6 illustrates the surface tension trend of the developed PILs. According to theory, the surface tension initially decreases as the concentration of the PILs increases, indicating a higher percentage of PIL molecules at the air–water interface. This area, known as the premicellar region [40], is characterized by the formation of premicellar aggregates at a specific concentration known as the critical aggregation concentration [41]. Following the premicellar region, the surface tension reaches a point called the critical micelle concentration, at which it ceases to change, signifying the formation of micelles. When the PIL level exceeds the cmc, the surface tension achieves a stable state and shows minimal variation, even with further increases in PIL concentration. This phase is referred to as the post-cmc region [42].

**Figure 7:**
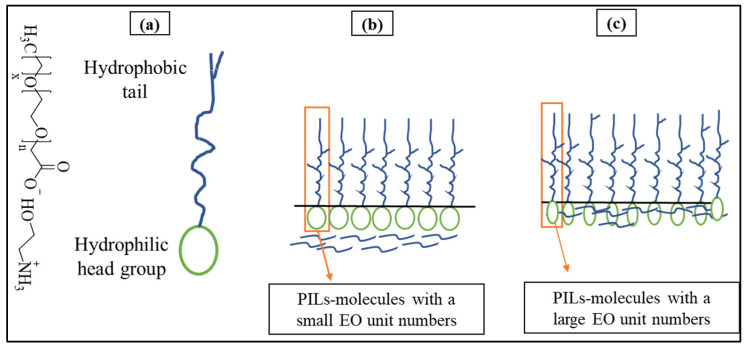
Mechanism of the aggregation process of PIL molecules in water mixture (**a**) structure of PILs, (**b**) PILs with small EO numbers, (**c**) PILs with large EO numbers.

In this study, it was found that the surface tension (*γ_cmc_*) of studied PILs decreased significantly from 72.8 mN/m to a plateau between 22.0090 and 37.6970 mN/m. Surface tension investigations for PILs are significant as they effectively depict the basic energy dynamics and interaction forces between cations and anions, providing an essential understanding of these PILs underlying physicochemical features and behavior in solution [43]. The synthesized PILs (PIL-360, PIL-460, PIL-690, and PIL-700) have similar ethanolamine as a Brønsted base and they differ in terms of the number of ethoxy (EO) units. Besides the change in the number of ethoxy units, the PIL-700 possesses an unsaturated chain to identify the role of double bonds in glycolic acid on aggregation behaviour. The surface tension results demonstrate a direct correlation between the number of EO additions and surface tension, as can be seen in Table 6. The relationship can be described as follows: when the number of ethoxy units increases, the surface tension also increases. The surface tension results in Figure 6 show that PIL-360 exhibits the lowest surface tension among the synthesized PILs. The data in Table 6 demonstrate a clear relationship between surface tension and the number of EO groups in the molecular structure of PILs. Specifically, a lower number of ethoxy units corresponds to lower surface tension. The surface tension values of the PILs follow the trend: PIL-360 < PIL-460 < PIL-690 < PIL-700. This behavior can be attributed to the fact that an increase in EO units enhances the polarity of PILs, leading to stronger interactions with water, which in turn increases the surface tension. On the other hand, PIL-700 molecules with an additional feature of unsaturation together with ethoxy units have comparatively large hydrophilic groups. These segments permit the molecules to have a looser arrangement at the interface between air and water, resulting in higher *γ_cmc_* values. The potential aggregation mechanisms of all PIL molecules take place in solution, as demonstrated in Figure 7 [43]. The presence of ethoxy groups reduces the thickness of the PIL monolayer at the air/water interface through two main mechanisms. First, the ether linkages in the ethoxy groups increase the overall hydrophilicity of PILs, leading to monolayer expansion and a decrease in thickness. Additionally, ethoxy groups alter the adsorption characteristics of PILs, affecting their interfacial behaviour [44,45].

The larger EO units undergo folding in space. As a result, the PIL molecules become smaller in size while maintaining the same length of hydrophobic chains. This is due to an increased number of EO, a more compact molecular structure, and a propensity for longer hydrophobic chains to bend [46]. Consequently, a significant number of -CH_2_ groups are visible on the uppermost region of the surface, leading to increased surface energy and a high *γ_cmc_*, as depicted in Table 6.

### 3.6. Thermodynamics Properties

The presence of PILs at the air/aqueous interface leads to a decrease in the surface tension [10]. The Gibbs adsorption equations were applied to compute various interfacial characteristics, such as the surface pressure at the cmc (*Π_cmc_*), surface excess concentration (*Γ_max_*), and the minimum area per molecule (*A_min_*), and the results are depicted in Table 6 [47]. The following formulas were used to calculate these surface properties.

(1) $$\Pi_{cmc}=\gamma_{0}-\gamma_{cmc}$$

(2) $$\Gamma_{max}=-\frac{1}{nRT}\;\left(\frac{d\gamma }{dInC}\right)$$

(3) $$A_{min}=\;\frac{10^{18}}{{N_{A\;}\Gamma }_{max}}$$

As shown in Table 6, an increase in EO groups in PIL molecules led to a reduction in surface excess concentration *Γ_max_* at the air/water interface and an increase in area per molecule *A_min_*. This suggests that including the EO group can considerably hinder PILs’ monolayer compactness at the interface between air and water. In addition, when the number of ethoxy groups increases the hydrophilic portion of the PILs molecules expands. This expansion leads to a comparatively loose configuration of the PIL molecules at the interface between air and water. Consequently, this reduces the total compactness of the monolayer at the interface. The same trend was observed by Chen et al. (2021), by using a series of anionic and non-ionic surfactants with different oxyethyl groups in the surfactant molecular structure for EOR. They observed a decrease in the surface excess concentration *Γ_max_* at the air/water interface, and an increase in the area per molecule *A_min_*, as the number of EO group in lignin polyether sulfonates molecules increased. This suggests that the presence of the EO group can greatly reduce the density of the surfactant monolayer at the air/water interface [46].

Additionally, the calculation of two key thermodynamic factors, such as the free micellization energy (Δ*G*°*_mic_*) and the free adsorption energy (Δ*G*°*_ads_*) can be done by using Equations (4) and (5).

(4) $$∆G_{mic}^{0}=RTIn\;cmc$$

(5) $$∆G_{asd}^{0}=∆G_{mic}^{0}-6.023\times 10^{-1}\times \Pi_{cmc}\times A_{min}\;$$

The values of Δ*G*°*_mic_* and Δ*G*°*_ads_* for all PILs with different EO unit numbers are negative, indicating that PILs spontaneously form micelles and adsorption of PILs at the air–water interface is spontaneous. The Gibbs free energy of adsorption is greater than the Gibbs free energy of micellization for all PILs indicating that the adsorption process is more prominent than micellization [48]. This statement suggests that the PIL molecules have a high ability to adhere to surfaces and develop a layer of adsorption in the solution. Subsequently, the layer that has been adsorbed becomes fully saturated and transforms into micellar aggregates. The thermodynamic properties results are consistent with the surface tension findings. Overall, the synthesized polymeric ionic liquids demonstrated exceptional surface and thermodynamic characteristics and a decreased cmc, which is a crucial factor to consider when choosing a chemical for use in oilfield applications.

## 4. Conclusions

This work effectively synthesized and examined polymeric ionic liquids (PILs) based on glycolic acid and ethanolamine containing different degrees of ethoxylation. These PILs were investigated for their potential use in oilfields. The successful synthesis of the PILs was validated by structural elucidation using NMR and FTIR. The PIL containing no ethoxy units (PIL-200) exhibits precipitation when dissolved in high-salinity brine; therefore, it is not suitable for oilfield applications. However, the PILs having ethoxy groups showed excellent solubility in low- to high-salinity brine and hence were evaluated further. Thermal gravimetric analysis revealed that all ethoxy-containing PILs have remarkable thermal stability, and the order of thermal stability was PIL-360 < PIL-460 < PIL-690 < PIL-700. The salt tolerance experiments demonstrated that the PILs retained their solubility and stability in high-salinity conditions, which is crucial for their effective use in oilfield applications. The surface tension studies showed that the PILs successfully decreased surface tension, resulting in a significant rise in surface area per molecule (*A_min_*) and a drop in surface excess concentration (*Γ_max_*) as the number of ethoxy groups increased. These results indicate that the presence of ethoxy groups greatly decreases the thickness of the PILs monolayer at the air/water interface. The thermodynamic study revealed that the PILs exhibit the spontaneous formation of micelles and adsorption at the water surface, with favorable free micellization and adsorption energies. Overall, the synthesized PILs demonstrated low cmc, reduced surface tension at cmc and thermodynamic characteristics, positioning them as attractive contenders for oilfield applications in challenging reservoir settings.

## Figures and Tables

**Table 1:** The salt composition of SSW and SFW.

Salt	Seawater (g/L)	Formation Water (g/L)
NaCl	41.042	150.446
CaCl_2_.2H_2_O	2.385	69.841
MgCl_2_.6H_2_O	17.645	20.396
Na_2_SO_4_	6.343	0.518
NaHCO_3_	0.165	0.487
Total TDS	67.58	241.688

**Table 2:** ^1^H-NMR data and peak description of PILs.

^1^H-NMR Data, *δ* in ppm, 600 MHz							
Polymeric Ionic Liquids (PILs)	Glycolic Acid					Ethanolamine	
	a	b		c	d	e	f
**PIL-200**	0.88	1.28	1.52	-	-	2.99	3.76
**PIL-360**	0.88	1.25	1.57	3.65	4.15	3.02	3.92
**PIL-460**	0.87	1.27	1.57	3.65	4.15	3.00	3.92
**PIL-690**	0.88	1.26	1.57	3.65	3.96	3.06	3.96
**PIL-700**	0.79	1.20	1.45	3.53	3.78	2.90	3.61

**Table 3:** Carbon NMR data and peak description of PILs.

Carbon NMR Data, δ in ppm, 150 MHz									
Polymeric Ionic Liquids (PILs)	Glycolic Acid							Ethanolamine	
	a	b				c	d	e	f
**PIL-200**	14.1	22.7	26.3	29.7	31.9	-	181.7	41.9	58.8
**PIL-360**	14.1	22.7	26.0	29.5	31.9	70.4	176.1	42.2	58.9
**PIL-460**	14.1	22.6	26.0	29.4	31.8	70.2	176.1	42.4	59.4
**PIL-690**	14.1	22.6	26.0	29.5	31.9	70.1	175.7	42.3	58.3
**PIL-700**	13.2	22.4	26.8	29.4	31.7	70.3	176.5	41.7	58.0

**Table 4:** FTIR results of PILs.

Polymeric Ionic Liquids (PILs)	FTIR Results				
	*υ*O-H	*υ_as_*C-H	*υ_s_*C-H	*υ*COO	*υ*OCO (Symm.)
**PIL-200**	3410	2920	2851	1531	-
**PIL-360**	3360	2921	2853	1586	1107
**PIL-460**	3406	2923	2854	1585	1099
**PIL-690**	3421	2920	2855	1585	1099
**PIL-700**	3410	2924	2854	1589	1099

**Table 5:** Solubility profile of the synthesized PILs.

PILs	DW	SSW	SFW
**PIL-200**	Cloudiness	Precipitation	Severe precipitation
**PIL-360**	Transparent	Transparent	Transparent
**PIL-460**	Transparent	Transparent	Transparent
**PIL-690**	Transparent	Transparent	Transparent
**PIL-700**	Transparent	Transparent	Transparent

**Table 6:** Surface properties of synthesized PILs.

Polymeric Ionic Liquids	cmc (mmol/L)	*γ_cmc_* (mN/m)	*Π_cmc_* (mN/m)	*Γ_max_* (mol/mm^2^)	*A_min_* (nm^2^)	*ΔG_mic_* (KJ/mol)	*ΔG_adv_* (KJ/mol)
**PIL-360**	0.031	22.009	49.991	4.582 × 10^−12^	0.362	−10.985	−17.555
**PIL-460**	0.022	25.322	46.678	4.277 × 10^−12^	0.388	−11.042	−17.292
**PIL-690**	0.018	33.013	38.987	3.471 × 10^−12^	0.394	−11.790	−18.554
**PIL-700**	0.002	37.697	34.303	3.218 × 10^−12^	0.474	−14.125	−19.132

## Data Availability

Data will be made available upon request.
